# Computer-Controlled Local Anesthesia Complication: Surgical Retrieval of a Broken Dental Needle in Noncooperative Autistic Paediatric Patient

**DOI:** 10.1155/2020/6686736

**Published:** 2020-11-10

**Authors:** Damian Chybicki, Małgorzata Lipczyńska-Lewandowska, Gaja Torbicka, Anna Janas-Naze

**Affiliations:** Department of Oral Surgery, Central Clinical Hospital, Medical University of Lodz, Poland

## Abstract

The article describes an unusual case of retrieval of 8 mm fragment of a broken 30-gauge 21 mm dental needle in a 6 y.o. noncooperative autistic male patient. The needle of a computer-controlled local anesthesia device was broken during an attempt to administer local anesthetic, in order to perform conservative treatment of teeth 55 and 54 by a pedodontist. Despite the fact that the patient was under nitrous oxide sedation, an unexpected movement of the patient occurred and resulted in needle breakage. Due to the lack of patient cooperation, the surgical retrieval of a broken needle was performed under general anesthesia as part of one-day surgery procedures. The purpose of the article is to emphasize careful decision-making in proper choice of dental instruments during treatment of noncooperative paediatric patients even under sedation and to suggest dentists to carry out treatment of such patients under general anesthesia.

## 1. Introduction

Autistic disorder is categorized in the *DSM-IV* (*Diagnostic and Statistical Manual of Mental Disorders, 4th Edition*), and it is characterized by abnormal emotional, social behavior, and linguistic development [1, 2]. The interest in autism has grown tremendously over the past twenty years. Knowledge and awareness of this disorder grow in all areas of public life, among parents and health professionals.

**S**killful dental treatment without pain is one of the basic principles of treating children in a dental office, especially those with behavioral disorders. This is an essential step towards obtaining and maintaining cooperation [3]. Children are very sensitive to unpleasant sensations in the dentist's chair, which determines their future attitude to treatment. Fear of dental treatment is common in children and affects 6% to 42% of young patients. This psychological condition can completely eliminate the child's willingness to cooperate with the dentist [4]. Various techniques, both pharmacological and nonpharmacological, have been developed to alleviate this negative attitude (e.g., distracting the child, atraumatic restorative techniques, and local anesthesia with a camouflaged syringe) [4, 5].

Local anesthesia has a significant role in pain control and the success of dental procedures. Although local anesthesia is intended to completely eliminate the pain during treatment, the traditional techniques of its administration are painful for the patients [6].

However, paradoxically, patients often fear pain caused by anesthetic injections more than pain from dental treatment itself [7]. Fear/anxiety-related behavior has long been recognized as the most difficult aspect of patient management and can undermine adequate dental care, especially in children [8]. Despite careful anesthetic procedures, dental local anesthesia can cause pain for a variety of reasons, for example, soft tissue damage during penetration of the oral mucosa by the needle, pressure, temperature and low pH of the anesthetic solution, and pain from the characteristics of the drug [7].

Several techniques have been introduced in order to reduce pain caused by the administration of local anesthesia, for example, topical anesthesia, distraction method, warming the anesthetic solution, regulation of the injection rate, buffering anesthetic agent, counterirritation, and local precooling with refrigerant spray [3]. Among the above-mentioned methods, topical anesthesia is the most widely used for reducing pain associated with needle insertion [3].

Computer-controlled anesthetic delivery systems have been developed in an attempt to attenuate or overcome patients' fear and anxiety of local anesthesia required prior to invasive procedures. According to the manufacturers of these electronic devices, it provides a precise injection flow rate and allows controlling the pressure exerted according to tissue resistance and density variations [8]. All these features are aimed at eliminating unpleasant sensations during the application of local anesthesia.

Both demanding cases and patients often present themselves to the dentist specialist, for treatment that requires highly specialized methods. One of these methods is nitrous oxide sedation. Nitrous oxide is a colorless gas with a sweetish taste so it is pleasant for children. It is an effective analgesic and anxiolytic agent that causes depression and euphoria in the central nervous system with negligible effects on the respiratory and cardiovascular system. For some patients, especially noncooperative children, the feeling of loss of control could be a problem, some patients find the nasal mask imprisoning and have the feeling of not breathing well [9]. This situation may result in unexpected and sudden movement of the patient and complications such as described in the article.

## 2. Case Report

**Figure 1:**
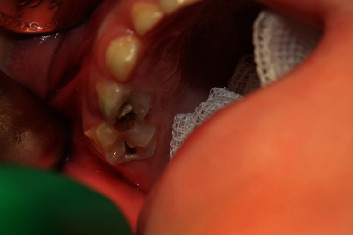
Intraoral view of operative region. Even though the needle is visible on the radiograph, it is not possible to firmly locate it intraorally.

A 6-year-old patient, suffering from a mild form of autism, who does not require any medications, was referred by a pedodontist to the Department of Oral Surgery, Medical University of Lodz, for removal of a broken needle from a computer-controlled local anesthesia device. The local anesthesia was performed a day before, due to a necessary conservative treatment of teeth 55 and 54. The circumstances of this situation were as follows: at first, the patient was sedated with nitrous oxide through a nasal mask; the next step was to administer local anesthetic with a computer-controlled local anesthesia device. The purpose was to eliminate, as much as possible, unpleasant sensations associated with appointment in a dental office and dental treatment. During administration of local anesthetic, unexpected movement by the patient occurred, which resulted in 30-gauge and 21 mm needle breakage and, consequently, total loss of the patient's cooperation. On the day of the patient's appointment in our Department, extraoral examination showed no pathologies. Intraoral examination revealed no symptoms of mucosa inflammation in the operative region; also, the broken needle was not visible. Teeth 54 had caries on its occlusal and distal surfaces, and tooth 55 had caries on its occlusal and mesial surfaces (Figure 1).

**Figure 2:**
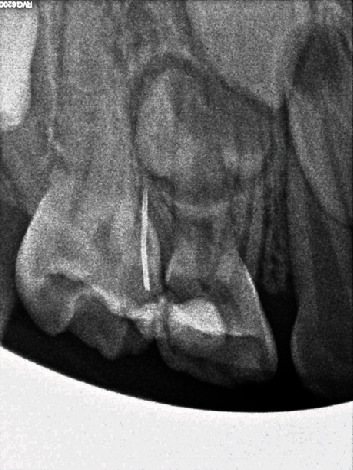
The periapical radiograph shows the exact location of a broken tool between teeth 55 and 54.

Based on clinical examination and radiological evaluation (Figure 2), the treatment plan included designing the correct surgical access to interproximal space between teeth 54 and 55 and retrieval of the broken needle. After presenting the parents with a detailed plan and obtaining mandatory permissions, the preoperative recommendations, medications, and laboratory blood tests were ordered (complete blood count, APTT), and the surgery was scheduled.

**Figure 3:**
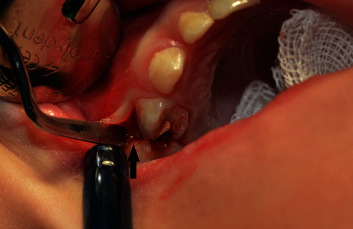
The broken needle is visible (black arrow) in periodontal ligament space of tooth 55 after flap reflection.

Under general anesthesia (short-term intravenous anesthesia with use of combination of 50 mg of propofol and 20 *μ*g of fentanyl with monitoring the saturation and heart rate), a sulcular incision along the gingival margin of teeth 55 and 54 was performed with scalpel blade No. 15 (Swann-Morton, Sheffield, England), and the flap was reflected with the Molt 9 periosteal elevator (Kohler Medizintechnik, Stockach, Germany) (Figure 3).

**Figure 4:**
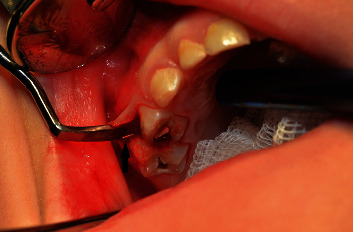
Surgical retrieval of a broken needle (black arrow).

**Figure 5:**
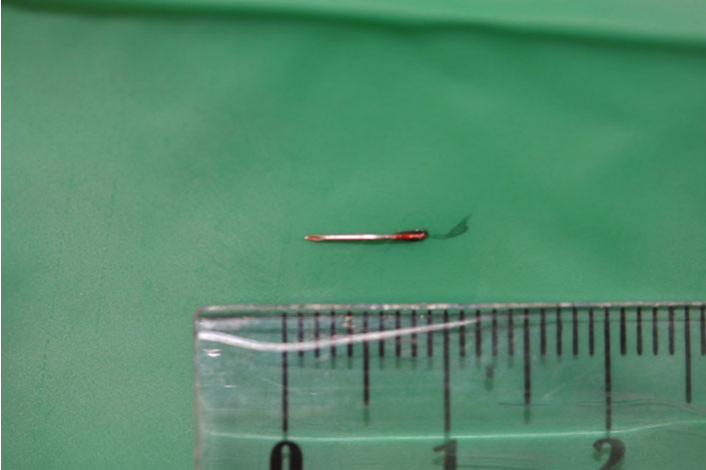
Removed needle, approximately 8 mm long.

The needle was removed from periodontal ligament space with a dental applicator (Figures 4 and 5).

The flap was repositioned, and simple interrupted sutures were performed (Novosyn 4/0, B. Braun, Germany). The patient regained consciousness in the recovery room and was discharged home. Postoperative antibiotic (150 mg of clindamycin every 8 hours) and analgetic (150 mg of ibuprofen depending on the needs, but not more than three times a day) were prescribed.

Follow-up examination was performed the day after surgery. Proper healing of the wound was observed, with no edema of the operative region and no pain. Sutures were left for spontaneous dissolution. Two weeks after surgery, the wound was completely healed. The patient did not report any complaints.

## 3. Discussion

Patients with autism should be treated with appropriate caution as their behavior in a dental office cannot be fully predicted; moreover, the oral health care of such patients can be complicated as they sometimes are not able to describe complaints about any dental problems they may be experiencing [2]. All these circumstances require a dentist to be flexible in their approach to such a patient, as well as to select the appropriate treatment tools for dental procedures. The main purpose is to eliminate pain, vibrations, and acoustic stimuli, which can worry an autistic patient.

Local anesthesia is the most common form of pain control in dentistry. Several different measures and various techniques and devices are used to attain local anesthesia in the mouth. Some of these methods, such as periodontal ligament injection, are unique to dentistry. Pain can occur during a variety of dental interventions, which involve dental surgery or stimulation of the dental pulp by cutting dentine. Common dental treatments performed in children causing pain, which can be prevented by using local anesthetic, include the placement of restorations and extraction of teeth [10].

Administration of local anesthesia is one of the most feared dental procedures by the patient. The pain perceived during local anesthesia administration in children is mitigated by various methods such as application of topical anesthetics [3], camouflaging of the syringe [5], distraction with audiovisual glasses [11], and counterstimulation. Vibration, pressure application, and precooling are different types of counterstimulatory measures to reduce pain perception during local anesthesia administration [12]. The new generations of dental anesthesia devices, which are designed to minimize unpleasant sensations during administration of local anesthetic, are computer-controlled local anesthesia devices. The flow of injection is adjusted to, among others, tissue resistance so the patient does not feel the expansion of anesthetic solution. Both children and adult patients can benefit from the advantages of these devices [8, 13]. Since the introduction of computer-controlled local anesthetic delivery systems, studies have compared its effectiveness to traditional methods of local anesthesia. Some of the cases found in literature have compared the pain of injection with the computer-assisted injection system to a conventional needle. Results from a majority of studies have favored the computer-assisted injection system [6].

However, despite all these measures, sometimes the dentist fails to achieve a satisfactory level of cooperation with a young patient. Then, it is possible to use nitrous oxide (laughing gas), to relax a stressed child and perform dental treatment with due care. During sedation, the reduction of physiological and psychological responses of the patient to surgery is obtained, without a loss of consciousness, collaboration, and protective reflexes; it is used to treat moderately anxious patients and allows for a calm and relaxed patient during therapy, with anterograde amnesia [9].

In extreme cases, when any cooperation with the child is impossible, the only solution is treatment under general anesthesia as it was described in this case report. This kind of treatment requires the presence of an anesthesiologist and the recovery room after dental procedure for the patient to regain consciousness. General anesthesia is defined as a controlled state of drug-induced loss of consciousness during which patients cannot be aroused, even by painful stimuli, and lose their protective reflexes. Independent ventilatory function is frequently inadequate, and cardiovascular function may be impaired [14, 15]. The indisputable advantage of treatment under general anesthesia is that almost every patient can undergo it, which means that, regardless of age and cooperation, it is possible to achieve therapeutic success.

In this case report, breakage of a 30-gauge 21 mm needle resulted from sudden movement of the patient. However, such complications can also be observed in adults whose behavior is calm. Other causes of needle breakage include bending the needle before injection, placing it in the tissues up to its base, and using needles with a diameter inappropriate for the anatomical situation [16]. The latter factor is of exceptional importance, as it results from the desire to minimize the pain of the injection, although it has been proven that the pain caused by the injection does not depend on the diameter of the needle. In addition, thinner needles are prone to cause more pain because the pressure applied on the syringe is much greater with a small gauge needle, so it is advisable to use a 27-gauge 21 mm needle, instead of a 30-gauge 21 mm needle, for young patients who have low pain threshold [17].

This article presents a case of a broken dental needle as a foreign body. Other foreign bodies that may be encountered in the oral cavity theoretically include almost all tools used by the dentist. There are cases that describe iatrogenic breakages of dental instruments during mandibular third molar surgeries: dental root elevator embedded into a subgingival caries of second mandibular molar [18], elevator tip, broken and buried in soft tissues [19], breakage of high-speed handpiece bur [20]. Patients themselves can also place various objects in the mouth that can break, especially into the canals of the teeth, as well as into interproximal spaces. Examples of these situations may be tips of metallic compasses, stapler pin, copper strip, and sewing needles [21].

Removal of foreign bodies from the oral cavity is very important as they can lead to chronic inflammation with the formation of fistulas and abscesses [21]. When breakage of any instrument occurs, the most important is communication between the patient and the dentist so the patient is aware of the condition of his body. If the dentist is not able to remove the separated instrument, then the patient should be referred to a more specialized medical center.

## 4. Conclusions

Dental treatment of autistic children should be carried out in specialized centers by experienced pedodontists. This is due to the fact that it is impossible to predict the child's behavior in various situations taking place in the dental office. As presented in this article, treatment in sedation with nitrous oxide, despite the child's initial cooperation, may result in serious complications such as needle breakage in oral tissues. It should be considered whether in cases of behavioral disorders such as autism treatment under general anesthesia is the most suitable option for the patient.

## Data Availability

The references used to support the findings of this case report are listed in References.
